# Environmental Constraints Guide Migration of Malaria Parasites during Transmission

**DOI:** 10.1371/journal.ppat.1002080

**Published:** 2011-06-16

**Authors:** Janina Kristin Hellmann, Sylvia Münter, Mikhail Kudryashev, Simon Schulz, Kirsten Heiss, Ann-Kristin Müller, Kai Matuschewski, Joachim P. Spatz, Ulrich S. Schwarz, Friedrich Frischknecht

**Affiliations:** 1 Parasitology, Department of Infectious Diseases, University of Heidelberg Medical School, Heidelberg, Germany; 2 Max Planck Institute for Metals Research, Department of New Materials and Biosystems, and University of Heidelberg, Department of Biophysical Chemistry, Stuttgart, Germany; 3 Max Planck Institute for Infection Biology, Department of Parasitology, Berlin, Germany; 4 Institute for Theoretical Physics and Bioquant, University of Heidelberg, Heidelberg, Germany; Faculdade de Medicina da Universidade de Lisboa, Portugal

## Abstract

Migrating cells are guided in complex environments mainly by chemotaxis or structural cues presented by the surrounding tissue. During transmission of malaria, parasite motility in the skin is important for *Plasmodium* sporozoites to reach the blood circulation. Here we show that sporozoite migration varies in different skin environments the parasite encounters at the arbitrary sites of the mosquito bite. In order to systematically examine how sporozoite migration depends on the structure of the environment, we studied it in micro-fabricated obstacle arrays. The trajectories observed *in vivo* and *in vitro* closely resemble each other suggesting that structural constraints can be sufficient to guide *Plasmodium* sporozoites in complex environments. Sporozoite speed in different environments is optimized for migration and correlates with persistence length and dispersal. However, this correlation breaks down in mutant sporozoites that show adhesion impairment due to the lack of TRAP-like protein (TLP) on their surfaces. This may explain their delay in infecting the host. The flexibility of sporozoite adaption to different environments and a favorable speed for optimal dispersal ensures efficient host switching during malaria transmission.

## Author Summary

Guidance of motile cells plays an important role during the life of a multi-cellular organism from early embryogenesis to the intricate interactions of immune cells during an infection. These migrations, like those of pathogens, can be directed by both chemical and physical cues. The malaria parasite needs to migrate immediately after being injected into the skin of the host by a mosquito bite. The malaria parasite forms deposited in the skin are called sporozoites. These must penetrate the dermis of the host to reach and enter a blood vessel. It is not clear if the sporozoites follow chemical cues or rely on the physical context of the environment. We show here, using *in vivo* imaging that sporozoites migrate along different paths in different skin environments. Introducing a novel assay for the study of cell migration in general we show that these *in vivo* paths can be largely recreated *in vitro* by placing sporozoites in a micro-patterned environment. This shows that environmental constraints are sufficient to guide sporozoite migration in the skin dermis. We further speculate that sporozoites have evolved to migrate at the fastest speed possible for efficient dispersal and show that a parasite lacking a surface protein has substantial defects in tissue dispersal and thus cannot efficiently infect the host.

## Introduction

Optical analysis of cell migration can be performed in simple cell culture assays, in reconstituted 3D-environments, tissue explants or in living animals [1], [2]. Clearly, the migratory patterns of cells have evolved in the context of the environment they maneuver in. Many cells, such as white blood cells, neurons, metastatic tumor cells or sperm cells as well as bacteria rely on a chemotactic response system to guide them towards their target [3]. Because chemotactic signals are usually subjected to large noise levels, in all of these cases cells follow stochastic search strategies based on different variants of a Brownian random walk [4]. However, a complex pattern of movement inside for example skin or lymphoid tissue can also arise in the absence of chemotactic signals due to stochastic elements in the movement-generating processes or structural constraints in the environment [5]. For example, naive T and B cells were shown by intravital two-photon microscopy to move inside lymphoid organs on independent and apparently random 3D-paths [6], [7]. Although intravital imaging is arguably a very powerful tool to observe cells in their natural environment, analysis of *in vivo* data can be hampered by a number of factors such as the micro-architecture of an organ, which can potentially mask a weak chemotactic effect [5], [8].

Here we analyze motile *Plasmodium* sporozoites, which are the forms of the malaria parasite injected into the dermis during a mosquito bite [9], [10]. They move rapidly in seemingly random paths and can invade blood or lymphatic vessels [10]. Sporozoite motility is essential for establishing an infection within the host [11]. It relies on an actin-myosin motor, which is placed just beneath the plasma membrane of the parasite and is linked to the substrate by trans-membrane proteins of the TRAP (thrombospondin-related anonymous protein) family to the substrate [12]. Different TRAP family proteins appear to play distinct roles during sporozoites adhesion and motility [13]–[21]. While we begin to understand the molecular basis of parasite motility, little is known about how *Plasmodium* sporozoites reach the blood vessels after they are injected into the dermis by a mosquito. In principle sporozoites could either follow chemotacic cues [22] or be guided by their physical environment. Curiously, sporozoites migrate on near perfect circular paths on 2D substrates, which extend to spirals in 3D gels [22], [23]. To migrate within the skin after a mosquito bite presents a formidable challenge to the parasite as the mosquito bite amounts to a strong disruption of the tissue structure (including haemorrhages) and as neutrophils rush to the side of infection to clear introduced pathogens [24]–[26]. It is thus not apparent how sporozoites could successfully detect and follow a chemotactic signal in such a situation. The aim of this study is therefore to assess if structural constraints in the skin are sufficient to create the characteristic motility patterns of parasites and guide them through the skin. We reasoned that micro-fabricated substrates featuring pillars of well-defined spacings and feature sizes, often used to investigate the mechanical activity of large vertebrate cells or molecular networks [27]–[30] could serve as adequate obstacle arrays to deflect motile sporozoites, which can be placed in-between the pillars. Applying a wide range of different obstacle geometries we found that parasites can be easily deviated from their circular motion observed on planar substrates. Imaging wild type sporozoites in the absence or presence of actin depolymerizing drugs and sporozoites lacking the TRAP family protein TLP (TRAP-like protein) [20], [21] followed by quantitative analysis of their movement patterns, allowed us to define and experimentally probe key parameters of sporozoite movement. Depending on the distance of the obstacles we found parasites predominantly moving in linear or meandering patterns, preferences also observed *in vivo* in different skin sites.

## Results

### Distinct sporozoite movement patterns in different skin environments

**Figure 1:**
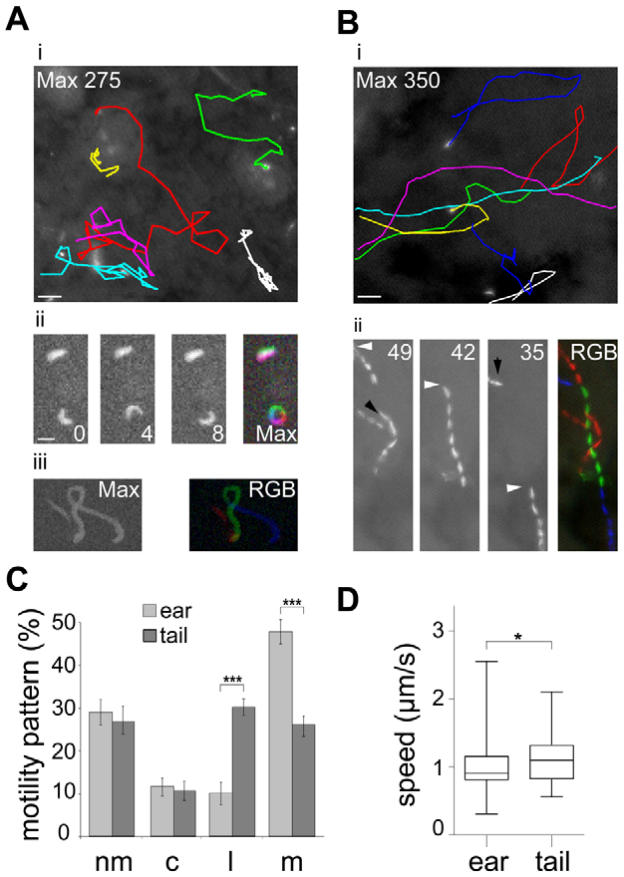
Different patterns of *P. berghei* sporozoite migration in the skin. (A) Sporozoites gliding within the skin of the ear. i: Colored tracks of 6 gliding sporozoites over 275 s (see Video S1) . Scale bar: 25 µm. ii: single frames and maximum fluorescent intensity projection of an immotile and a circular gliding sporozoite. Scale bar: 10 µm. iii: projection of a meandering sporozoite (left: max projection over 82 s; right: pseudo-colored track (RGB) of three different time periods of the same acquisition). Time is indicated in seconds. (B) Sporozoites gliding within the skin of the tail. i: Colored tracks of 8 gliding parasite over 350 s (see Video S1). Scale bar: 25 µm. ii: projection of three different time periods within one acquisition and the pseudo-colored merge (RGB) of these projections. White arrowheads point to the same parasite during the acquisition; black arrowheads point to the paths of other parasites. Time is indicated in seconds. (C) Automatically detected motility patterns of sporozoites imaged in the skin of the ear (n = 589) and tail (n = 164) showing a preference for meandering and linear movement patterns in the ear and tail, respectively, *** indicates P<0.001, motility patterns: nm = non motile; c = circling; l = linear; m = meandering. (D) Speed plot showing the median speed distribution of all tracked parasites imaged at 0.2 Hz in the dermis of ear or tail; whiskers and box indicate 97.5% and 50% of all values, respectively; horizontal bar: median (ear: 0.91 µm/s; tail: 1.10 µm/s; * indicates P<0.05).

To quantitatively assess the behavior of sporozoites inside their natural environment after transmission we carried out *in vivo* imaging of parasites expressing cytoplasmic GFP [31] inside the skin of the ear pinnae and the tail of living mice (Figure 1). In both sites sporozoite movement appeared to follow different patterns. While sporozoites in the ear rarely moved on linear paths, those injected into the skin of the tail were frequently seen to move for tens of seconds over several dozen of micrometers on almost linear trajectories (Figure 1A, B). Next we classified parasite movement according to the changing angles between subsequent positions of parasite tracks as movements in a linear, circular or meandering fashion (see Material and Methods). Additionally, parasites were categorized as non-motile when they were attached to the substrate and did not travel forward. Since parasites can vary their movement pattern over time we did not classify each single parasite but plotted the fraction for each pattern over the whole population. This analysis revealed that the fraction of non-motile sporozoites and circular moving sporozoites in the two distinct bite sites were similar at around 30% and 12% of the total observed time points from 589 and 164 sporozoites examined in the ear and tail, respectively (Figure 1C). The fraction of sporozoite movements that occur in a linear fashion was higher in the tail compared to the ear (P<0.001) (Figure 1C). Conversely, the fraction of meandering movement was higher in the ear than in the tail (P<0.001) (Figure 1C). Sporozoites moving in the tail moved about 20% faster than those in the ear (Figure 1D).

**Figure 2 ppat-1002080-g002:**
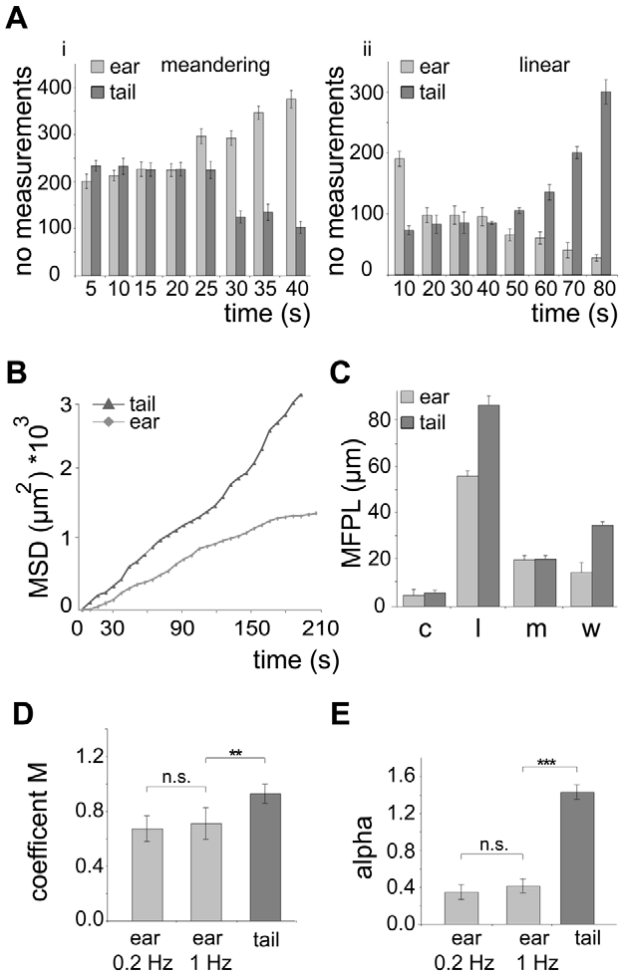
Quantification of *in vivo* sporozoite motility patterns. (A) Number of measurements for meandering (i) and linear (ii) movements binned over different times showing that in the ear and tail meandering and linear movements, respectively, are more persistent. (B) Plot of mean square displacement (MSD) over time comparing sporozoite tracks from ear and tail (further information Figure S1A, B). (C) Mean free path length (MFPL) of parasites gliding *in vivo*. The weighted values reflect the MFPL according to the percentage of parasites undergoing one particular movement pattern. Motility patterns: c = circling; l = linear; m = meandering, w = weighted (further information Figure S1C,D). (D, E) Motility coefficient M (D) and the actual scaling exponent α (E) of parasites gliding *in vivo*. For the parasites gliding inside the ear no difference was detected between movies acquired at an imaging rate of 0.2 Hz and 1 Hz. Parameters were calculated from the MSD plots in Figure 2B (further information in Figure S1G, H, I).

We next determined key parameters of sporozoite dispersal. As already apparent from *in vivo* projections (Figure 1A, B), individual sporozoites moving inside the skin of the ear maintained meandering motility for longer periods of time than sporozoites inside the skin of the tail (Figure 2Ai). Similarly, sporozoites moving in a linear fashion in the tail move both longer and further before changing their direction than sporozoites inside the ear (Figure 2Aii). To determine whether parasite movement follow a random walk pattern [8], we plotted and analyzed their mean square displacement (MSD) over time (Figure 2B–E, Figure S1). MSD of sporozoites moving in the ear becomes flatter after about 3 minutes of movement indicating a restricted random migration as would be expected from dispersal in the presence of obstacles (Figure 2B). In fact most biological systems do not obey normal diffusion due to internal processes or external factors like obstacles that influence their motion patterns, leading to so called sub-diffusion [32]. The MSD plot for movement in the tail shows a slight increase over time implying a more linear migration as shown in Figure 1C and 2B. Since a linear migration pattern should result in a MSD curve following a 2^nd^ order polynomial function, the nearly perfect fitting (R^2^ = 0.99) to the tail data confirms the predomination of linear patterns (Figure S1B). Other patterns should follow a first-order regression, which was indeed shown by the nearly perfect 1^st^ order fitting to parasite migration in the ear where meandering patterns dominate (Figure S1B). Furthermore, the sporozoites in the tail disperse even more rapidly than would be expected from the difference in speeds (Figure 1D, 2B).

Parasites moving in a linear fashion also showed the longest mean free path length (MFPL), i.e. the average distance covered in one type of pattern before changing into another type of pattern (Figure 2C, Figure S1A–D). As a consequence, parasites delivered to the tail covered longer distances than parasites in the ear when moving in a linear fashion. Moreover, weighting the MFPL with the percentage of the observed specific motility pattern showed that parasites moved longer distances in the tail (∼34 µm) compared to parasites moving in ear tissue (∼13 µm) before changing their motility pattern (Figure 2C).

To confirm our quantitative imaging analysis, we finally calculated key parameters describing dissemination from the origin, the motility coefficient M [8] and the actual scaling exponent α [33] from the MSD plots (Figure S1G–I). Alpha is dependent on the possibility and frequency for cell deflection influenced by the presence of obstacles (Figure S1H). M is a measure for how fast cells displace from their starting positions during a random walk process (Figure S1I). As expected, M was higher for sporozoites moving inside the dermis of the tail than in the dermis of the ear (Figure 2D) reflecting the higher speed of parasites in the tail. Parasites moving inside the tail show an α larger than 1, due to their dominant linear movement pattern in this tissue (Figure 1C, 2E). Parasites moving inside the ear show an α smaller than 1 (Figure 2E). The latter reflects a sub-diffusion behavior of sporozoites indicating that their motility might be influenced by the presence of deflecting obstacles [34].

### Deflecting sporozoite movements *in vitro*

**Figure 3 ppat-1002080-g003:**
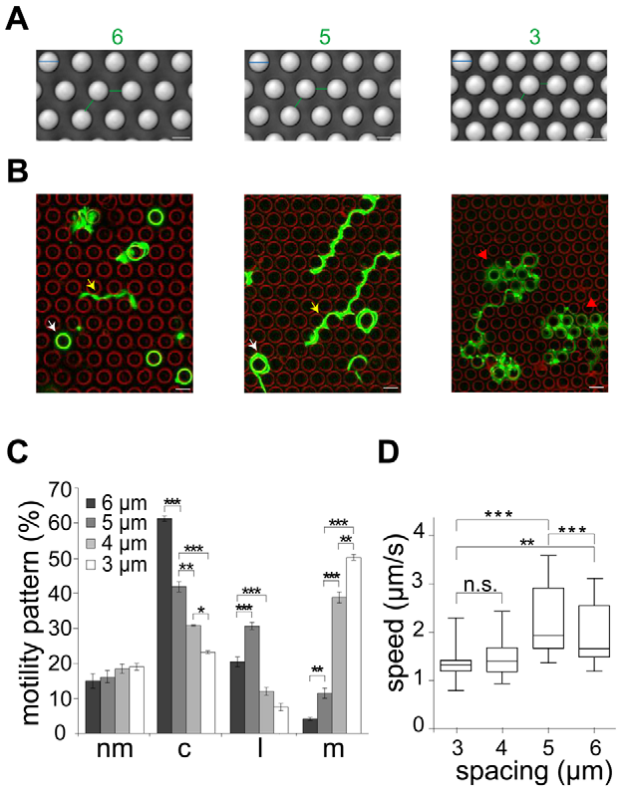
Different sporozoite migration patterns in micro-structured obstacles arrays. (A) DIC images of different micro-fabricated obstacle arrays in top view. Pillar diameter (cyan lines): 10 µm. Numbers and lines (green) indicate pillar-to-pillar distance in µm, also valid for panel B. Scale bars: 10 µm. (B) Merge of sporozoite trajectories (green) within different obstacle arrays (pillar circumferences in red). White arrowheads point to selected circular patterns. Yellow and red arrowheads point to linear and meandering movement patterns, respectively (see Video S2). Scale bars: 10 µm. (C) Influence of pillar-to-pillar spacing on the distribution of motility patterns in the different pillar substrates. At least 600 sporozoites were tracked and analyzed from at least 3 independent experiments for each different substrate; motility patterns: nm = non motile; c = circling; l = linear; m = meandering. (D) Whiskers plots of speed distribution of all parasites tracked *in vitro*. The horizontal bar indicates the median, the box represents the lower and upper quantile, the whiskers correspond to the 2.5 to 97.5 percentile of the data set.

**Figure 4:**
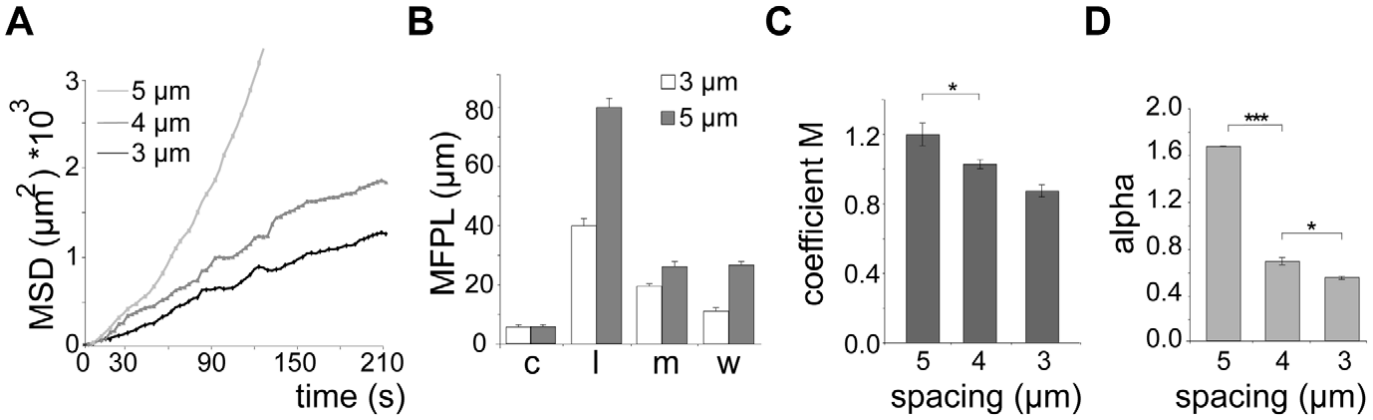
Quantification of sporozoite motility patterns in obstacle arrays. (A) Mean square displacement (MSD) plot over time of sporozoites moving in 3, 4 and 5 µm arrays. The MSD values of circling parasites were excluded (see Figure S1E, S1F). (B) Mean free path length (MFPL) of parasites gliding in the indicated arrays. Weighted values consider the percentage of parasites performing one particular movement pattern. Motility patterns: c = circling; l = linear; m = meandering. (C, D) Motility coefficient M (C) and actual scaling exponent α (D) of parasites gliding in arrays of the indicated pillar-to-pillar distances. Parameters were calculated from the MSD plots in Figure 4A.

To test if deflection of sporozoites contributes indeed to their overall migration paths, we utilized micro-fabricated pillar substrates as defined obstacle arrays. These were generated with individual pillar diameters of 10 µm and pillar-to-pillar distances from 2 to 6 µm (Figure 3A, Figure S2A, B). At least 600 sporozoites (from at least triplicate experiments) were tracked and analyzed for each different obstacle array if not stated otherwise. Visual inspection showed that decreasing pillar-to-pillar distance altered the movement pattern of the sporozoites (Figure 3B). Pillar contact and sporozoite speed was uniformly distributed over a parasite population with normal length (10.5 to 13.5 µm). Sporozoites placed between pillars spaced 6 µm apart mainly moved in circles around the pillars. In a non-constrained environment like on glass around 50% of the observed parasites turn in circles. On arrays with 6 µm or 7 µm pillar-to-pillar distance the percentage of circling sporozoites was even increased to around 60%. However, with decreasing pillar-to-pillar distances, fewer parasites go in circles with a higher occurrence of complex movement patterns increasing from ∼20% (34 of 164 for 6 µm) up to ∼70% (124 of 172 for 2 µm) (Figure 3C, S2C). Curiously, the number of linear movements increased from around 20% to around 30% when the pillar-to-pillar distance was reduced from 6 to 5 µm (P<0.001, n = 642 total sporozoites for 6 µm and n = 671 for 5 µm) but then sharply dropped when the distance was further decreased to 4 µm or 3 µm (n = 638 for 4 µm and n = 52 of 651 for 3 µm) (Figure 3C). In contrast, the number of meandering trajectories increased from around 5% to around 50% with decreasing pillar-to-pillar distance. Only ∼20% of sporozoites did not move (Figure 3C, Table S1). The highest median speed and fastest dispersal was found for sporozoites moving mainly on linear tracks on substrates with 5 µm pillar-to-pillar distance (5 µm arrays) (Figure 3D, Figure 4A, Figure S2D). Interestingly, sporozoites moving in 3 and 4 µm arrays showed a MSD plot comparable to sporozoites moving in the skin of the ear pinnae (Figure 2B and 4A, Figure S2E, F).

Similar to the situation in the skin (Figure 2C), parasites moving in a linear fashion in pillar arrays also showed the longest mean free path length (MFPL) (Figure 4B, S2D). Weighting the MFPL with the percentage of the observed specific motility pattern (Figure 4B) showed that parasites in 5 µm arrays had an overall longer MFPL (∼28 µm) than those in 3 µm arrays (∼10 µm) (Figure 4B, S2D). The motility coefficient M decreased with decreasing pillar-to-pillar distance with values from 1.2 µm^2^/s to 0.9 µm^2^/s (Figure 4C) due to higher mean velocities of sporozoites moving in a linear fashion in 6 µm and 5 µm arrays (Figure 3C, 3D and 4C). Similarly, α also decreased with pillar spacing, being larger than 1.5 in 5 µm arrays and less than 0.8 in 3 or 4 µm arrays (Figure 4D). As the increasing density of obstacles lead to an α<1 for parasite dispersal, this indicated that the presence of obstacles caused a sub-diffusion behavior of sporozoites.

### Sporozoite speed is optimized for migration through dense obstacle arrays

**Figure 5:**
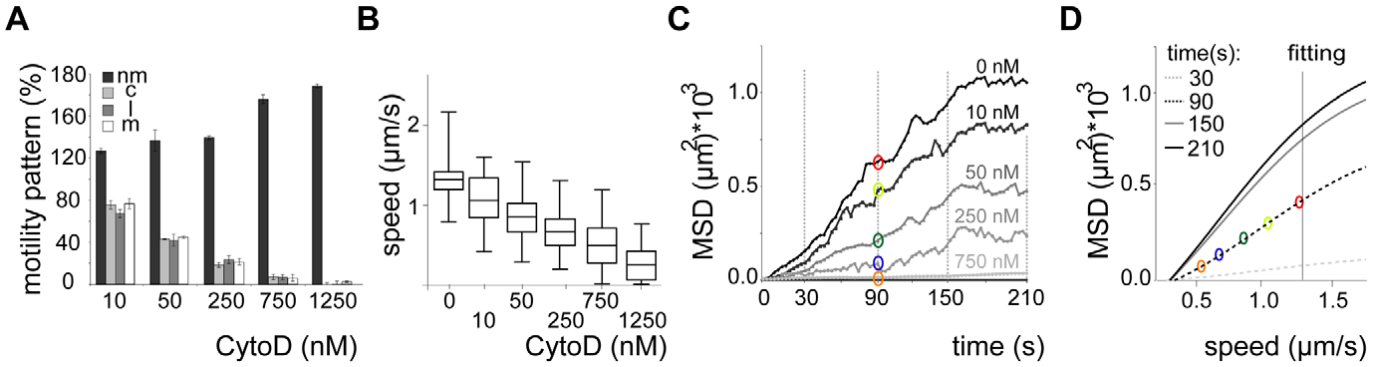
Dispersal of sporozoites under the influence of an actin filament-disrupting drug. (A, B) Automated detection of sporozoite movement patterns normalized to the control (0 nM cytoD = 100%) (A) and median speed distribution in a whiskers plot (B) from parasites imaged in the presence of increasing concentrations of cytochalasin D in 3 µm arrays. More than 150 sporozoites were tracked for each drug condition. Motility patterns: nm = non motile; c = circling; l = linear; m = meandering. (C) MSD over time for motile sporozoites imaged after adding increasing concentrations of cytochalasin D as indicated. Grey lines and colored circles indicate the distinct time points and the respective MSD values, respectively, as plotted in (D). (D) Fitted data of MSD at distinct time points from (C) versus the average speed of the sporozoite population under the varying concentrations of cytochalasin D from (B). Colored circles correspond to the MSD values extracted at 90 s as an example (C).

**Figure 6:**
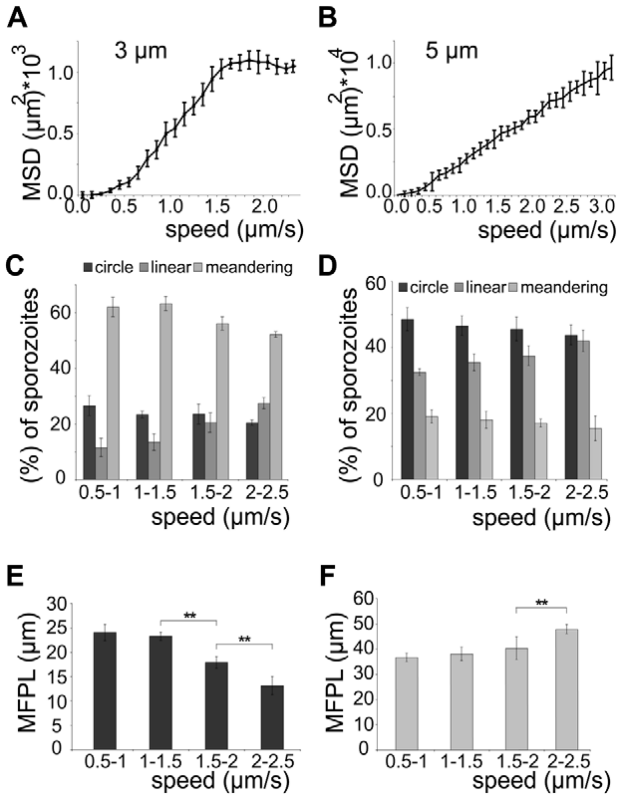
Sporozoite speed is optimized for efficient spread. (A, B) MSD values after 210 s of observation for individual sporozoites moving in 3 µm (A) or 5 µm (B) arrays plotted over their averaged speed. More than 300 sporozoites were analyzed for each array. (C, D) Sporozoite migration patterns for all parasites moving at the indicated speeds in 3 µm (C) and 5 µm (D) arrays. (E, F) Weighted mean free path length (MFPL) for all sporozoites migrating in 3 µm arrays (E) decreases with increasing speed, while for those migrating in 5 µm arrays (F) it increases at the fastest speeds.

We next probed how the speed of sporozoites is related to their dispersal in different arrays. To modulate speed we applied cytochalasin D (cyto D), which inhibits motility in a dose-dependent fashion [35]. Importantly, the application of increasing concentrations of cyto D affected the average speed of the parasites by mainly increasing the proportion of non-moving parasites while decreasing all movement patterns uniformly (e.g. increase of around 20% for 10 nM cyto D, n = 131) (Figure 5A, 5B). As expected with decreasing speeds drug treatment also caused a decrease in MSD (Figure 5C). We next plotted the MSD at certain time points against the average speed with which sporozoites were moving (Figure 5D). This showed a linear dependency at early times of movement and at low speeds but the MSD appeared to reach a plateau at about 1.3 µm/s. This value corresponds closely to the average speed of sporozoites observed *in vitro* as well as *in vivo* [10], [13], [23], [35]. As there is always a large range of sporozoite speeds within a population of parasites we refined our analysis by plotting the average speed for individual sporozoites from this experiment against the MSD at the end point (Figure 6A). The MSD over speed plot reaches a plateau phase for sporozoites moving in 3 µm arrays, while it increases linearly for those moving in 5 µm arrays (Figure 6A, B). Importantly the fraction of sporozoites moving in a circular or meandering fashion does not increase with increasing sporozoite speed (Figure 6C) and can therefore not cause the observed plateau phase (Figure 6A). In 5 µm arrays the linear increase of the MSD to speed plot is due to the increasing proportion of linear movers at higher speed (Figure 6D). This suggests that a difference in MFPL could cause this change. Indeed, the MFPL in 3 µm arrays decreases with increasing speed, while in 5 µm arrays it increases (Figure 6E, F). From this set of data it can be speculated that in 3 µm but not in 5 µm arrays sporozoites achieved an optimal compromise between energy expenditure for speed and dispersal in the environment.

### Parasites lacking TLP fail to disperse efficiently

Finally, we wondered if we could explain *in vivo* observations on efficient malaria transmission by comparing dispersal behavior of wild type (WT) and mutant sporozoites in pillar arrays. Sporozoites lacking the proteins SPECT-1, SPECT-2 and CelTOS, the surface phospholipase *Pb*PL and the TRAP family adhesin TLP (TRAP-like protein) have all been shown to be impaired in their capacity to cross the dermis [20], [21], [24], [36]–[38]. All mutant parasite lines display a deficiency in migration through cells (transmigration). However, only for one mutant sporozoite line, *tlp(-)* this deficiency could be correlated with impaired gliding locomotion *in vitro* [19]. *tlp(-)* sporozoites were shown to move at a slightly lower rate on their circular trajectories than WT sporozoites and detached more frequently from the substrate [19].

To investigate *tlp(-)* sporozoites in a quantitative manner in obstacle arrays, we first generated a *tlp(-) P. berghei* line (strain ANKA) that expresses green fluorescent protein in the parasite cytoplasm under control of the EF1alpha promoter [39] (Figure S3A, S3B). Sporozoites from isolated clones from this GFP-*tlp(-)* line displayed the same *in vitro* motility features as the previous non-fluorescent *tlp(-)* sporozoites (strain NK65), such as reduced average speed and speed distribution over a parasite population [19], [21] (Figure S3C, S3D). GFP-*tlp(-)* sporozoites also showed similar phenotypes in transmission experiments (Table 1). Importantly, GFP-*tlp(-)* sporozoites injected by mosquito bite or subcutaneously showed a delay of about one day in the emergence of blood stages, while sporozoites injected intravenously did not (Table 1). This finding further strengthens the hypothesis that *tlp(-)* sporozoites show a defect in crossing the skin to invade blood capillaries [19]–[21]. We then tracked over 300 GFP-*tlp(-)* parasites for each investigated pillar array (from at least three independent experiments).

**Table 1:** Transmission of *GFP-tlp(-)* sporozoites.

Genotype	No. of animals/No. of infected animals				Prepatent period (days)			
	i.v.	s.c.	b.b.-10	b.b.-5	i.v.	s.c.	b.b.-10	b.b.-5
**WT**	6/6	6/6	5/5	5/5	4.5	4.7	4.3	4.5
**GFP-** ***tlp(-)***	5/5	6/6	5/5	5/5	4.4	6.0	5.3	5.7

WT, wild type (ANKA GFP); GFP-*tlp(-)* (ANKA); i.v., intravenous injection of 100 salivary gland sporozoites; s.c., subcutaneous injection of 1.000 salivary gland sporozoites; b.b., 10 or 5 infectious mosquito bites; prepatent period is the time until the first detection of an erythrocytic-stage parasite in Giemsa-stained blood smears after i.v., s.c. or b.b. infection.

**Figure 7:**
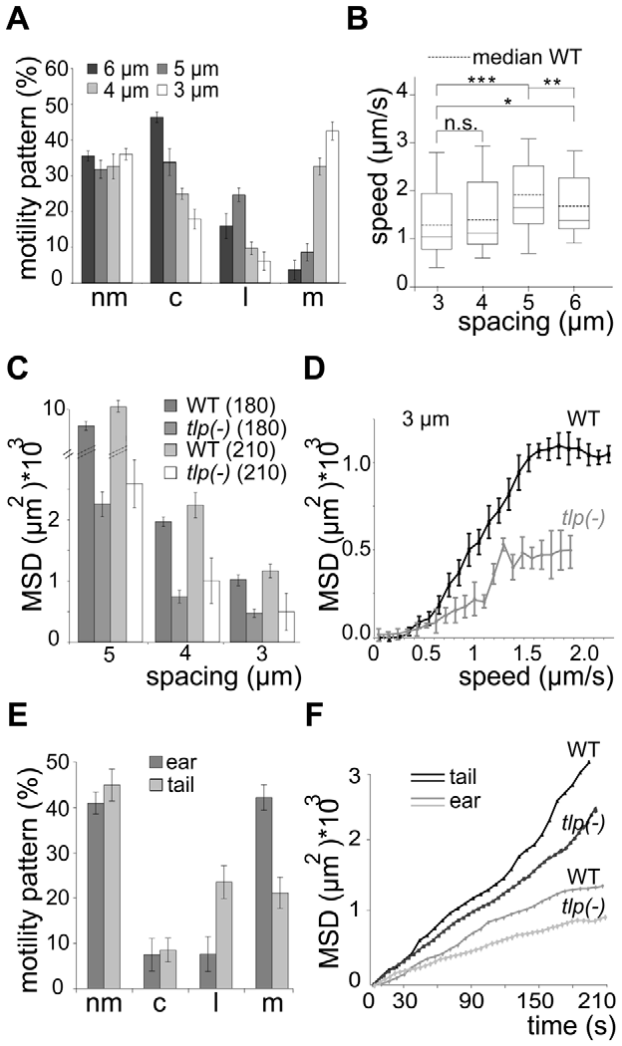
Slow dispersal of sporozoites lacking TLP. (A) Automated quantification of motility patterns in the different obstacle arrays similar to Figure 3C. At least 600 sporozoites were tracked and analyzed in at least 3 independent experiments for each different array (see Video S2). motility patterns: nm = non motile; c = circling; l = linear; m = meandering. (B) Median speed distribution of all GFP-*tlp(-)* parasites tracked *in vitro*; horizontal bar: median; dotted horizontal bar: median of the wild type sporozoites as taken from Figure 3D. (C) Comparison of MSD after 180 and 210 seconds of movement of wild type and GFP-*tlp(-)* sporozoites. Note that fewer sporozoites could be analyzed for 210 seconds (52) of movement than for 180 seconds (over 300). (D) MSD plotted over averaged sporozoite speed of WT and GFP*-tlp(-)* sporozoites moving in obstacle arrays of 3 µm pillar-to-pillar distance, respectively. (E) Automatically detected motility patterns of GFP-*tlp(-)* sporozoites imaged in the skin of the ear (n = 274) and tail (n = 92) showing an increase of non motile parasites compared to WT (Figure 1C); motility patterns: nm = non motile; c = circling; l = linear; m = meandering. (F) Plot of mean square displacement (MSD) over time comparing WT and *tlp(-)* sporozoite tracks from ear and tail.

When monitored in the obstacle arrays GFP-*tlp(-)* sporozoites showed a similar overall change of migration patterns as WT sporozoites. The numbers of circling and meandering sporozoites in obstacle arrays were directly and inversely correlated to the pillar-to-pillar distance, respectively (Figure 7A). As reported for motility on obstacle-free, two-dimensional glass slides [19], about 10% fewer GFP-*tlp(-)* sporozoites were attached to the substrate and consequently fewer sporozoites were moving in the respective patterns (Figure 7A, e.g. for 3 µm array an increase of non motile parasites from around 20% (n = 651 total sporozoites) to around 33% (n = 342). Of these, fewer GFP-*tlp(-)* sporozoites were moving for long periods of time being often interrupted by non-motile phases. Analysis of Stop (speed<0.3 µm/s) and Go (speed>0.3 µm/s) phases revealed an increase of Stop periods for GFP-*tlp(-)* parasites (Figure S4A, B), which were twice as long in time as those for WT parasites (Figure S4A, C). The Stop phases did not alter significantly between the different motile modes. Like WT sporozoites, GFP-*tlp(-)* sporozoites also moved fastest in obstacle arrays with 5 µm pillar-to-pillar distance (Figure 7B) but at slightly lower overall speeds than wild type sporozoites. Accordingly their mean square displacement was lower than that of wild type sporozoites (Figure 4A, 7C, Figure S3E).

Few GFP-*tlp(-)* sporozoites migrated for more than 3 minutes as they detached more frequently than the wild type. The MSD of GFP-*tlp(-)* sporozoites was reduced by 40–50% compared to the wild type (Figure 7C). Curiously, however, the MSD over speed plot revealed that GFP-*tlp(-)* sporozoites moving at the same average speed as wild type sporozoites dispersed less (Figure 7D, Figure S3F). This finding shows that average speed is not the sole variable determining sporozoite dispersal in complex environments. Finally, we tested whether the impaired migration revealed *in vitro* can also be observed in the dermis of ear and tail of mice. *In vivo* imaging of GFP-*tlp(-)* sporozoites (n_ear_ = 274, n_tail_ = 92) from 20 transmission experiments showed that the overall fraction of non motile parasites is increased by around 12% compared to the WT (Figure 1C, 7E). Accordingly, the fraction of motile parasites was uniformly decreased with a consistently higher percentage for linear or meandering movers in tail and ear, respectively. The MSD of motile GFP-*tlp(-)* parasites was significantly decreased compared to the WT (Figure 2B, 7F). The MSD curves, however still followed the characteristic 1^st^ or 2^nd^ order polynomial function for parasite migration in ear and tail (Figure 7F). Finally, both speed and MFPL of migrating GFP-*tlp(-)* sporozoites were also slightly reduced compared to WT (Figure 1D, 2C, Figure S3G, H) for sporozoites migrating in the tail but not in the ear. Interestingly, while WT sporozoites migrating in the tail were faster then those in the ear, no such difference was found for GFP-*tlp(-)* sporozoites (Figure S3G).

## Discussion

### Chemotaxis versus environmental guidance

It was previously suggested that sporozoites isolated from the mosquito hemolymph but not salivary gland-derived sporozoites were attracted to extracts of salivary glands [22]. However, whether naturally transmitted sporozoites, i.e. those from the salivary glands, are chemotactic or not is currently not known. We first investigated migration in different *in vivo* environments. Sporozoites migrating in the dermis of the tail more often showed movement in linear trajectories than sporozoites migrating in the skin of the ear. Conversely, sporozoites in the dermis of the ear pinnae showed more random migration patterns (Figure 1) indicating increased restriction (Figure 2). The observed difference between the migration patterns of sporozoites in different tissues may arise from chemical cues. Alternatively the patterns might be due to a difference in tissue architecture or be derived from a mix of both influences. The architecture of the tail skin is indeed known to be different from that of all other tissues [40]. Thus motility could be influenced differently by varying interactions of sporozoites with cells, fibers or other structures in the respective tissue environments of the ear and tail. Similarly, it was recently shown that thymocytes exhibit varying motion patterns within specific regions in the medulla, but not in the cortex of the thymus [41].

As the calculated key dispersal parameters from *in vivo* imaging suggest that sporozoites follow a sub-diffusion type of random walk, we hypothesized that sporozoites can be guided through the environment by being deflected from obstacles. To test this, we utilized micro-patterned obstacle arrays [27]–[30]. This analysis showed that sporozoites in some arrays adopted complex movement patterns that partially resembled those seen in the skin (Figure 1–4). Analysis of sporozoites moving in obstacle arrays revealed strikingly similar MSD, MFPL and alpha values to sporozoites moving in the skin of the ear pinnae, for 3 or 4 µm arrays or the tail, for 5 µm arrays (Figure 1–4). Interestingly, the same trend is also observed for speed, which is highest for sporozoites moving in the tail and those moving in 5 µm arrays.

These data together show that movement patterns of motile cells can be influenced by changes in the distances of obstacles. In the case of sporozoites, differences in *in vivo* migration trajectories can thus be partially reconstructed *in vitro* using obstacle arrays. We suggest that changes in parasite trajectories are rather due to the reflection of the motile sporozoites by different obstacles then to an actively altered migration pattern, which might be expected in order to optimize the chance of finding a blood capillary. As there are no chemical gradients present in the obstacle arrays, our data suggest that the complex patterns of sporozoites observed *in vivo* could also be explained by the presence of structural constraints imposed by the environment. However, our data does not allow us to exclude possible contributions to the modulation of parasite migration *in vivo* by chemical stimuli or 3D structures like collagen fibers, which are missing in the obstacle arrays. Parasite gliding could additionally be modulated by responses towards stimuli based on e.g. immune responses or gradients build up over longer time periods. On the other hand, *in vivo* imaging did not reveal a clear target-oriented rush of the parasites towards blood capillaries. The pillar experiments thus clearly indicate the importance of considering the role not only of chemical signals but also of physical constraints for sporozoite migration. Future research is necessary to elucidate if chemo- or durotaxis based signals could additionally influence sporozoite migration.

### Optimal sporozoite speed evolved for *in vivo* environments

While fast-moving mammalian cells like lymphocytes migrate at less than 0.1 µm/s, *Plasmodium* sporozoites move at 1–2 µm/s in the skin, a remarkably fast speed for substrate-dependent locomotion [6], [10]. Our data allowed us to probe if this rapid sporozoite movement is optimized for an efficient spread in the skin or if sporozoites just move as fast as they possibly can. This question cannot be answered by analysis of *in vivo* data, as only few fast moving sporozoites can be imaged and tracked for a long enough time *in vivo*. Also currently no methods are available to rapidly and conditionally manipulate sporozoite speed *in vivo*. As an alternative and based on the good correlation between sporozoite movement patterns *in vivo* and in pillar arrays we could manipulate sporozoite speed *in vitro* (Figure 5, 6).

Modulating the speed with the actin-disrupting drug cyto D revealed that, as expected, dispersal is directly dependent on the average speed of the migrating parasite population (Figure 5). Plotting the displacement (MSD) of every single parasite against its average speed revealed a linear relationship at low speed for 3 µm arrays (Figure 6A).

Unexpectedly, at higher average speed (between 1.5 and 2.5 µm/s) the spread of parasites reached a plateau phase in 3 µm arrays, while it continued to increase in pillar arrays that favored a linear migration pattern (5 µm arrays) (Figure 6B). As no major shift in movement pattern was observed for faster moving sporozoites, the majority of sporozoites gliding at higher speed in 3 µm arrays still displayed a meandering pattern (Figure 6C). The observed plateau phase is therefore likely due to the shorter mean free path length of fast moving parasites, which encounter more obstacles during the same period of time as slow moving ones (Figure 6C). Thus, in environments such as the skin where parasites undergo meandering migration, it can be speculated that the parasite would need considerably more energy in order to further increase its spread if moving faster than 1.5 µm/s. Indeed, *in vivo* ∼90% of the sporozoites move slower than 2 µm/s (Figure 1D), suggesting that sporozoites have adapted to the environmental constraints that limit the mean free path length. We therefore speculate that sporozoites have evolved to reach an optimal level of dispersal relative to energy expenditure for speed.

### Gliding-impaired parasites fail to disperse efficiently

We also investigated parasites lacking the plasma membrane protein TLP, which appears to be important for sustained rapid motility on circular tracks *in vitro* [19], for exiting the skin and penetrating and/or growing within the liver [20], [21]. These parasites dispersed much less in all tested obstacle arrays than WT parasites due to a shift to a larger number of non-motile parasites and a slower migration speed (Figure 6). However, unlike for parasites treated with cytoD, the percentage of non-motile phases increased due to more frequent and longer resting phases of motile parasites. This is likely due to the adhesion impairment of *tlp(-)* sporozoites [19]. Strikingly, even *tlp(-)* parasites moving at the same average speed as WT parasites showed a smaller MSD (Figure 7B, 7D). This can be explained by the different range of instantaneous speeds for the two different populations (Figure 3D, 7B). GFP-*tlp(-)* sporozoites show a broader range of e.g. 0.5 to 2.8 µm/s compared to 0.8 to 2.3 µm/s for wild type sporozoites (Figure 3C, 7B). This shift is likely due to the change in the adhesive capacity of the parasites in the absence of TLP [19]. The high maximum instantaneous speeds of GFP-*tlp(-)* sporozoites in the 3 and 4 µm arrays fall into the plateau phase of the MSD over speed plot and thus limits their contribution to dispersal (Figure 6A). This shows that the average speed is not always directly linked to parasite dispersal and suggests that a careful dissection of parameters describing motility is necessary for the understanding of a parasite line showing a subtle motility deficient phenotype. This also suggests, that the adhesion capacity of sporozoite surface proteins, as exemplified by TLP, influences their efficient dispersal in complex environments. Finally, the decreased dispersal rate could contribute to the significant increase in the time needed for *tlp(-)* sporozoites to cause a blood stage infection if injected by mosquitoes or into the subcutaneous tissue (Table 1). This would suggest that the motility defects seen in *tlp(-)* sporozoites compared to WT reveal that TLP functions mainly in crossing the skin barrier after transmission. To probe this hypothesis we performed *in vivo* imaging of GFP-*tlp(-)* parasites. These parasites were less motile and showed only a slight reduction in speed but dispersed less than wild type parasites (Figure 7). This shows that the mutant line is migration impaired *in vivo* as well as *in vitro*. In comparison, however (Figure 7A–D and 7E–F), the reduction of dispersal of *tlp(-)* sporozoites was not so pronounced *in vivo* as on pillar arrays. In arrays with obstacles spaced 5 or 3 µm apart the MSD was decreased by around 50 to 70% (from around 10*10^3^ µm^2^ to around 3*10^3^ µm^2^ for 5 µm and from around 1*10^3^ µm^2^ to around 0.5*10^3^ µm^2^ for 3 µm arrays, respectively) (Figure 7C). In the skin a decrease of around 25% in MSD was revealed (e.g. from around 1*10^3^ µm^2^ to around 0.8*10^3^ µm^2^ for ear) (Figure 7F). The less distinct influence of the loss of TLP for *in vivo* migration could be explained by the difference between the quasi-2D environment of the pillar substrate and the complex 3D environment of the dermis. For example, a 3D surrounding could better compensate for adhesive defects as parasites detaching from one side could adhere at the other side almost simultaneously, which is obviously not possible to the same degree in pillar arrays. Additionally, factors induced by the inflammatory response to the mosquito bite or the mosquito saliva were not present in pillar arrays. We currently do not know if these factors influence parasite motility. Also, the stiffness of a PDMS obstacle array is clearly different to the more elastic skin environment. Concerning TLP function, we can also not rule out that this protein also functions in blood vessel entry. However, despite these shortcomings, we could successfully use obstacle arrays to reveal differences in parasite migration patterns that resemble *in vivo* migration much better than those observed on plain 2D surfaces, where sporozoites simply glide in circles.

Micro-patterned obstacle arrays can be used to deepen our understanding of how parasites travel trough the skin in order to reach the capillary system and establish infection. The characterization of a genetically defined parasite mutant further illustrates how micro-patterned obstacle arrays, in combination with transmission experiments, could be used to rapidly screen for motility defects during host switch. Whole cell assays, such as the one described herein, in combination with diverse small compound libraries offer a potential for anti-malaria lead substance discovery [42], [43]. Importantly, these arrays could also be adapted for use with other cells to similarly check for defects in migration behavior of a variety of cell types and pathogens.

## Material and Methods

### Ethics statement

All animal experiments were performed concerning FELASA category B and GV-SOLAS standard guidelines. Animal experiments were approved by German authorities (Regierungspräsidium Karlsruhe, Germany), § 8 Abs. 1 Tierschutzgesetz (TierSchG).

### Production of micropillar substrates

We first designed a master mask using a home-written program called Phyton and converted it with the mask-writer software DWL-66 (Heidelberg Instruments). The individual pillars were arranged in hexagonal patterns to assure the distances of 2 to 6 µm between pillars in any direction (Figure S2A). The master mask was produced by ML&C Jena. The PDMS micropillar substrates were then made by photolithographic and replicate molding techniques as described in [29]. To allow wetting of the hydrophobic pillar substrates they were first treated chemically with Extran, an alkaline solution (Merck, Germany) in a 1∶10 dilution in H_2_O for 20 minutes while gently shaken. Afterwards the structures were washed three times for 10 minutes in distilled water. A silicon flexiPERM chamber (GreinerBioOne) was used for imaging parasites. The chamber surrounds the micropillar substrate and maintains a stable environment without flow (Figure S2B). For the motility studies on pillar substrates the sporozoites in RPMI containing 3% BSA (bovine serum albumine) were added to the flexiPERM chamber surrounding one pillar field. For the cytochalasin D treatments the parasites were preincubated with the different drug concentrations for 15 minutes.

### *Plasmodium* sporozoites and *in vitro* imaging

Fluorescent *P. berghei* (NK65) or GPF-*tlp(-)* (ANKA) sporozoites [31] (see section: Generation of GFP-*tlp(-)* sporozoites) were produced and prepared as described [35], [44]. Comparison of parasite motility *in vitro* showed no difference between NK65 and ANKA strain parasites (Figure S3). Imaging of sporozoite motility on the different obstacle arrays was performed on an inverted Axiovert 200M Zeiss microscope using the GFP filterset 37 (450/510) at room temperature. Images were collected with a Zeiss Axiocam HRM at 1 Hz using Axiovision 4.8 software and a 10×, 25× or a 40× Apoplan objective lens (NA = 0.25 for 10×). A single DIC (differential interference contrast) image of the substrate was taken before and after the analysis to merge the PDMS pillars with the time-lapse series of motile sporozoites recorded in the GFP channel. For the motility analysis, a time lapse of one image per second for a total of 300 frames was taken. For each obstacle array at least three different region of interest (ROIs) were recorded. For each substrate the experimental setup was repeated several times on different days with sporozoites harvested between 17–21 days after mosquito infection. Thus the motility parameters of at least 600 sporozoites for each pillar array were recorded. The DIC image and the GFP movie were overlaid and analyzed with ImageJ (http://imagej.nih.gov/ij/index.html). For visualization, the pillars were illustrated in red and the maximum intensity projections of the parasite trajectories in green (Figure 3B, Figure S2B).

### Intravital imaging

NMRI mice (Charles River) were anaesthetized by intraperitoneal injection of 70 µl ketamin/xylazin (200 µl/50 g) and the ear pinnae or the tail were exposed to infected mosquitoes inside a gauze-covered beaker as described [10], [13]. Mosquitoes were infected either with wild type (NK65) GFP fluorescent parasites [31] or GFP-*tlp(-)* parasites (ANKA) (see section: Generation of GFP-*tlp(-)* sporozoites). For each experiment mosquitoes were allowed to probe for 1–3 minutes at the exposed site before the mouse was placed on the microscope table of an inverted microscope. Parasites inside the skin were filmed using a GFP filterset either on an inverted widefield Axiovert 200M Zeiss microscope (using Axiovision 4.6 software and a 25× LCI Plan-Neofluar objective (NA 0.8)) or a PerkinElmer UltraView spinning disc confocal unit on an inverted Nikon TE 2000-E microscope (using PerkinElmer Ultraview Software and a 20× objective (PlanFluor multi-immersion, NA 0.75). Images were acquired at 0.2 or 1 Hz. The mouse was kept at a stable temperature using a preheated incubation chamber or a heating blanket.

### Generation of GFP-*tlp(-)* sporozoites

The plasmid used to generate the *tlp(-)* parasites [21] has been transfected into a *Plasmodium berghei* ANKA strain expressing cytoplasmic GFP [39] using standard transfection methods [45]. Clonal lines were obtained by limited dilution into 15 recipient NMRI mice. Genotyping of recombinant parasites was performed by gDNA extraction and integration PCR with the same primer combination as used by Heiss et al [21] (Figure S3A). Mosquitoes were infected with GFP-*tlp(-)* parasites and sporozoites isolated from infected mosquitoes as described [44]. To validate that the *tlp* gene was deleted in *GFP-tlp(-)* parasites transcript detection was performed by RT-PCR from total RNA obtained from gradient-purified schizonts or salivary gland-associated sporozoites (Figure S3B). GFP-*tlp(-)* and, as controls, WT parasites were used for RNA isolation and reverse transcription. cDNAs were synthesized from 2 µg of total RNA using Retroscript (Ambion). Reference pools (−) were obtained in the absence of reverse transcriptase. For detection of *TLP* transcripts we used primers PbTLPfor_1 and PbTLPrev_1 [21] and *Pb*aldolase primers (Aldolase_for 5′ TGTATTTAAAGCTTTACATGATAATGG 3′; Aldolase_rev 5′ TTTTCCATATGTTGCCAATGAATTTGC 3′, expected size: ∼450 bp) for transcript controls.

### Transmission experiments

4–6 C57Bl/6 mice were infected either intravenously (i.v.) with 100 WT (ANKA GFP) or GFP-*tlp(-)* sporozoites (ANKA), subcutaneously (s.c.) with 1,000 parasites or by bite (bite) using 10 or 5 mosquitoes per animal. The infected mosquitoes were pre-selected based on their GFP signal in the midgut one day prior to the transmission experiment and kept in a standard insectary incubator (Sanyo incubator) without the normally present sugar pad. Giemsa-stained smears from tail blood were monitored daily. The prepatent period is the time until the first detection of an erythrocytic-stage parasite in Giemsa-stained blood smears after infection.

### Data analysis

The parasites were either tracked manually using the ImageJ manual tracking plugin or with an automated tool, ToAST [35]. Designation of movement as either circling or complex was first performed manually by differentiation of circling parasites and parasites that were not turning in stable circles ( = complex pattern). For an automated analysis we generated a MATLAB script, which distinguishes between attached, circular or linear moving or meandering parasites according to the angular change during motility with the following constraints: motile sporozoites that changed their angle between 0 and 12 degrees from one frame to the next were classified as moving in a linear fashion. For angles between 12 and 45 degrees and >45 degrees the sporozoites were classified as moving in a circular and meandering fashion, respectively. Parasites moving slower than 0.3 µm/s were assigned as non motile. Using the MATLAB script, parasites were automatically classified and the speed, the mean square displacement (MSD), the actual scaling exponent α and the motility coefficient M were determined for motile parasites. Random walk patterns were analyzed by plotting the average mean square displacement from the origin of the motility against time [8]. The MSD was calculated for motile parasites moving faster than 0.3 µm/s [35] and in linear and meandering patterns, excluding circling parasites (Figure S1C, S1D). The slope of the linear regression on a logarithmic MSD plot corresponds to α while M can be derived from the offset [33] (Figure S1G). Linear regression was performed using MATLAB. The visualization of the trajectories was also performed in MATLAB.

### Statistical analysis

589 sporozoites in the ear and 164 sporozoites in the tail tissue were tracked and analyzed from over 25 transmission experiments (Figure 1). At least 600 sporozoites were tracked and analyzed for each different pillar array (Figure 3). Between 150 and 200 sporozoites were counted for the manual analysis (Figure S2C). For each drug treatment we tracked and analyzed more than 150 parasites (Figure 5). We tracked over 300 GFP-*tlp(-)* parasite for each investigated pillar array. GraphPad Prism was used for graphing and statistical analysis. Mean and standard deviations were plotted for each graph if not stated otherwise and Students t-test was performed. For not normally distributed unpaired data (*in vivo* data) the Mann-Whitney test was used (Figure 1D, 3D, 7B, S3G). The range of whiskers plots indicates the 2.5–97.5% percentile, the box includes 50% of all values and the horizontal bar shows the median. The threshold alpha of the p value was set to 0.05 (*), 0.01 (**) and 0.001 (***).

## Supporting Information

Figure S1 Quantitative analysis of cell and sporozoite displacement. (A) Three different cell trajectories D1, D2 and D3 are shown (grey). The displacement at a certain time point t (D(t) = D) is the direct distance a cell has traveled between the common start point and the coordinates at the time point t (“air line distance”, orange dotted lines). The free path length (FPL, grey lines) is the distance covered by a moving particle for each step from the start point (“real traveled path”). To quantify average displacements and path lengths of a large number of particles/cells the mean square displacement (MSD) and mean free path length (MFPL) are calculated. Particles can cover the same path length (fixed MFPL) but are less far (D3 = lower MSD) or further away (D1 = higher MSD), from the start point (black dot) than D2. (B) 1^st^ order regression or 2^nd^ order polynomial fitting to one data set for parasite migration in the ear or tail showing a very good fitting (R^2^<0.99). Since linear migration patterns dominate for tail migration over time the regression behaves as 2^nd^ order polynomial. Other patterns predominantly result in first order regressions thus giving a best fitting for migration in the ear where meandering patterns dominate. (C) Cells can be equally far away from the starting point (black dot) (fixed MSD) but have traveled on differently long paths in order to go there (variable MFPL, L3 shorter MFPL than L2 than L1). (D) Track plots of 7 sporozoites migrating *in vivo* inside the ear and the tail. Individual tracks are shown in different colors and were arranged so they originate at the same x-y position to visualize parasite spread. The absolute time is indicated in seconds. (E) Mean square displacement (MSD) plot over time of two sporozoites turning in circles with higher (blue) and lower speed (red). Individual perfectly circling parasites show no net increase in mean square displacement as they have the same start and end point during one circle. During one circle the MSD oscillates between 0 and maximum distance (half circle). (F) Mean square displacement (MSD) plot over time of 20 (blue), 50 (green) and 150 (black) circling sporozoites. Although individual parasites have no mean square displacement, the MSD of many sporozoites eventually reaches a plateau phase at around 500 µm^2^. This is due to the broad variation of circling since the diameter of the circle and the speed can vary between 8 to 12 µm and 1–2 µm/s. For this reason we only plot MSD from motile (average speed over 0.3 µm/s) sporozoites that do not move in a circular fashion (>30% meandering and/or linear motility). (G–I) The key parameters describing dissemination from the origin are the motility coefficient M (µm^2^/s) and the actual scaling exponent α. Most biological systems do not obey unrestricted diffusion like passive particles in solution because internal processes or external factors like obstacles influences the motion patterns [8], [34]. In this so called anomalous diffusion situation, the MSD is given by MSD(Δt) = 4DΔt^α^, where D is the diffusion coefficient also named motility coefficient M [8], α the actual scaling exponent [33] and Δt the time lag between two positions. (G) The standard method to test for abnormal diffusion is to find α and M through a linear fit to logarithmic scaling of the MSD plot. Logarithmic plotting of the MSD over time reveals a near linear curve (black) showing that a linear relationship between log(MSD) and log(t) can be assumed. Therefore, M and α are derived by linear regression (blue line) to log(MSD(Δt)) = αlog(Δt)+log(4M) [34]. An actual data set is given exemplarily (a 3 µm pillar array, black dots and curve). Fitting quality is given by R-squared (R^2^), where R = 1 indicates perfect fitting. The R^2^-values for our total data sets are: ear 0.934, tail 0.8523, 3 µm: 0.8934, 4 µm: 0.91275, 5 µm: 0.8702. The slope of the fit is α and the offset b yields the motility coefficient M = 10^b^/4. If obstacles dominate, one usually obtains a subdiffusion type of abnormal diffusion characterized by the MSD growing not linearly with the time t but characterized by t^α^, with α<1. (H) Conceptualizing the actual scaling exponent α. Two situations of cell trajectories are given. Fixed MFPL and variable MSD (left) or vice versa (right). α depends on the possibility and frequency of how often the cell (or particle) is deflected. More deflection can result in lower α (α3 is smaller than α2, which is smaller than α1). (I) Conceptualizing the motility coefficient M. Two situations of cell (or particle) trajectories are shown. As the motility coefficient (M) can be calculated from the offset b (panel S1G) it is a measure of how fast an object displaces from its starting position during a random walk. Left: at a fixed time point t_x_ M1 is higher than M2 resulting in longer MFPL, at time point t_y_ M1 and M2 are the same (resulting in same total MFPL). This can only be reached when M2 is higher than M1 between time point t_x_ and t_y_ in order for the object to travel the same overall distance. Thus, M is connected to speed since the object on trajectory 2 has to speed up between t_x_ and t_y_ to finally cover the same MFPL. Right: If the MFPL is fixed, the time t has to vary to reach a difference in M. Traveling the same distance at different M (M1>M2) results in reaching the set MFPL (on 1 trajectory) at an earlier time t_1_ compared to the later time t_2_ for the particle on trajectory 2. Thus, M is dependent on speed and cannot be totally decoupled from MFPL or MSD.
(TIF)

Figure S2 Micro-fabricated obstacle arrays. (A) DIC images of different micro-fabricated obstacle arrays (top view). Pillar diameter (cyan lines) is fixed to 10 µm while pillar-to-pillar distances are varied from 2 to 6 µm (green lines and numbers). Scale bars: 10 µm. (B) (i) Scheme of the experimental setup. The flexiPERM chamber (light grey) surrounds the obstacle array (dark grey) on a glass slide (white) and is imaged with a 10× objective (black). (ii) Cartoon of an obstacle array showing obstacles (red and orange) and a sporozoite (green) in 3D (upper panel) and a top view corresponding to the image in panel A and to Figure 3. The arrow indicates the movement of the sporozoite around a pillar. (C) Manual quantification of sporozoite movement patterns within different obstacle arrays of different pillar-to-pillar spacing. Between 150 and 200 sporozoite trajectories were evaluated for each pattern. All bars show mean ± standard deviation. (D) Track plots of 15 sporozoites migrating in 3 and 5 µm arrays. Individual tracks are shown in different colors and were arranged so they originate at the same x-y position to visualize parasite spread. The absolute time is indicated in seconds. (E) DIC images of different micro-fabricated obstacle arrays (top view). Pillar diameter (blue line) can be varied from 4 to 14 µm (here 8 µm, 10 µm and 12 µm are shown) while pillar-to-pillar distances can be varied from 2 to 7 µm (here 3 µm arrays are shown). Scale bars: 10 µm. (F) MSD over time plot for migrating sporozoites in arrays with different pillar diameters (8; 10 and 12 µm) and pillar-to-pillar spacing fixed to 3 µm. Parasite migration is independent from a pillar diameter between 8 µm to 12 µm.
(TIF)

Figure S3 Generation, evaluation and analysis of GFP-*tlp(-)* parasites. (A) Replacement-specific PCR analysis. In the replacement strategy cartoon the grey lines indicate binding of test primers for WT, *tlp* and integration of the *dhfr/ts* gene. PCR analysis reveals the specific disruption of *P. berghei tlp* locus. The successful replacement event is verified by diagnostic PCR primers [21]. Test 1 and 2 primer combinations can only amplify a signal after successful replacement and integration (see schematic replacement strategy). Absence of the wild type-specific signal from GFP-*tlp(-)* parasites confirms the purity of the clonal population. *tlp(-)*1 and 2: recombinant *tlp(-)* clones; parental: parental line with a mixed population of wild type (WT) and *tlp(-)* parasites. (B) RT-PCR. Absence of *TLP* transcripts in *tlp(-)* parasites. cDNA from WT or *tlp(-)* late-blood stages (blood) or sporozoites (spz) was amplified in the presence (+) or absence (−) of reverse transcriptase (RT) with *PbTLP*-specific primer combinations. As loading controls, RT-PCRs with *Pb*aldolase-specific primers were added. gDNA: genomic DNA from WT or clone *tlp(-)*-1 parasites. (C) Manual analysis showing that the GFP-*tlp(-)* sporozoites display the same movement patterns as *tlp(-)* parasites: na: not attached; a: attached; w: waving; ccw: counter-clockwise moving sporozoites. Note that there are about 10% fewer CCW moving GFP-*tlp(-)* sporozoites than WT sporozoites. (D) Gliding GFP-*tlp(-)* sporozoites move in the same manner as *tlp(-)* sporozoites. Analysis was performed as described in [19]. (E) MSD plots of WT and GFP-*tlp(-)* sporozoites moving in obstacle arrays of 3, 4 and 5 µm pillar-to-pillar distance. (F) MSD plotted over average sporozoite speed of WT and GFP-*tlp(-)* sporozoites moving in obstacle arrays of 5 µm pillar-to-pillar distance, respectively. (G) Speed plot showing the median speed distribution of all tracked GFP-*tlp(-)* parasites imaged in the dermis of ear or tail. The dotted line marks the WT median value (Figure 1D). Statistical significances: WT ear vs. *tlp(-)* ear: n.s.; WT tail vs. *tlp(-)* tail: P<0.05 *; WT tail vs. *tlp(-)* ear: n.s.; WT ear vs. *tlp(-)* tail: P<0.05 *; *tlp(-)* ear vs. *tlp(-)* tail: n.s.). (H) Mean free path length (MFPL) of GP- *tlp(-)* parasites gliding *in vivo*. The weighted values reflect the MFPL according to the percentage of parasites undergoing one particular movement pattern. Motility patterns: c = circling; l = linear; m = meandering, w = weighted.
(TIF)

Figure S4 Stop and Go phases of parasite migration in obstacle arrays. (A) Example of single WT and *tlp(-)* sporozoites moving in a meandering (m) or linear (l) pattern. Speed (µm/s) is plotted over time. To analyze Stop phases all time points with parasite speeds lower than 0.3 µm/s were scored. The arrows point on exemplary Stop phases with a duration of 1 (red), 2 (orange), 3 (light orange) or 4 (yellow) frames. These durations were added up for the plots in (B and C). (B) Quantitative analysis of wild type or *tlp(-)* parasites turning in circles (c, n = 12/11), moving linear (l, n = 14/15) or meandering (m, n = 16/15) are plotted in respect to the total percentage of Stop (stop) and Go (move) phases. (C) Quantitative analysis of the duration for a stop phase as measured in time-frames from 0.5 Hz image acquisitions and plotted for WT and *tlp(-)*, respectively.
(TIF)

Table S1 Movement patterns listed according to environment. Non moving parasites (∼20% in arrays, ∼30% *in vivo*) make up the missing percentage to 100%.
(DOC)

Video S1 The movie shows sporozoites gliding inside the skin of the ear pinnae or tail overlaid with the colored tracks resulting from manual tracking using ImageJ.
(MOV)

Video S2 The movie shows an overview of sporozoites (green) gliding in a 4 µm array; a meandering sporozoite (green) gliding in a 3 µm array; a linear moving sporozoite (green) gliding in a 5 µm array and a *GFP-tlp(-)* sporozoite moving in a 4 µm array (pillar circumferences are shown in red).
(MOV)
