# Supplementary figures and images for: Environmental Constraints Guide Migration of Malaria Parasites during Transmission
Source: PLoS Pathog. 2011 Jun 16;7(6):e1002080. doi: 10.1371/journal.ppat.1002080 (PMC3116815; doi:10.1371/journal.ppat.1002080)

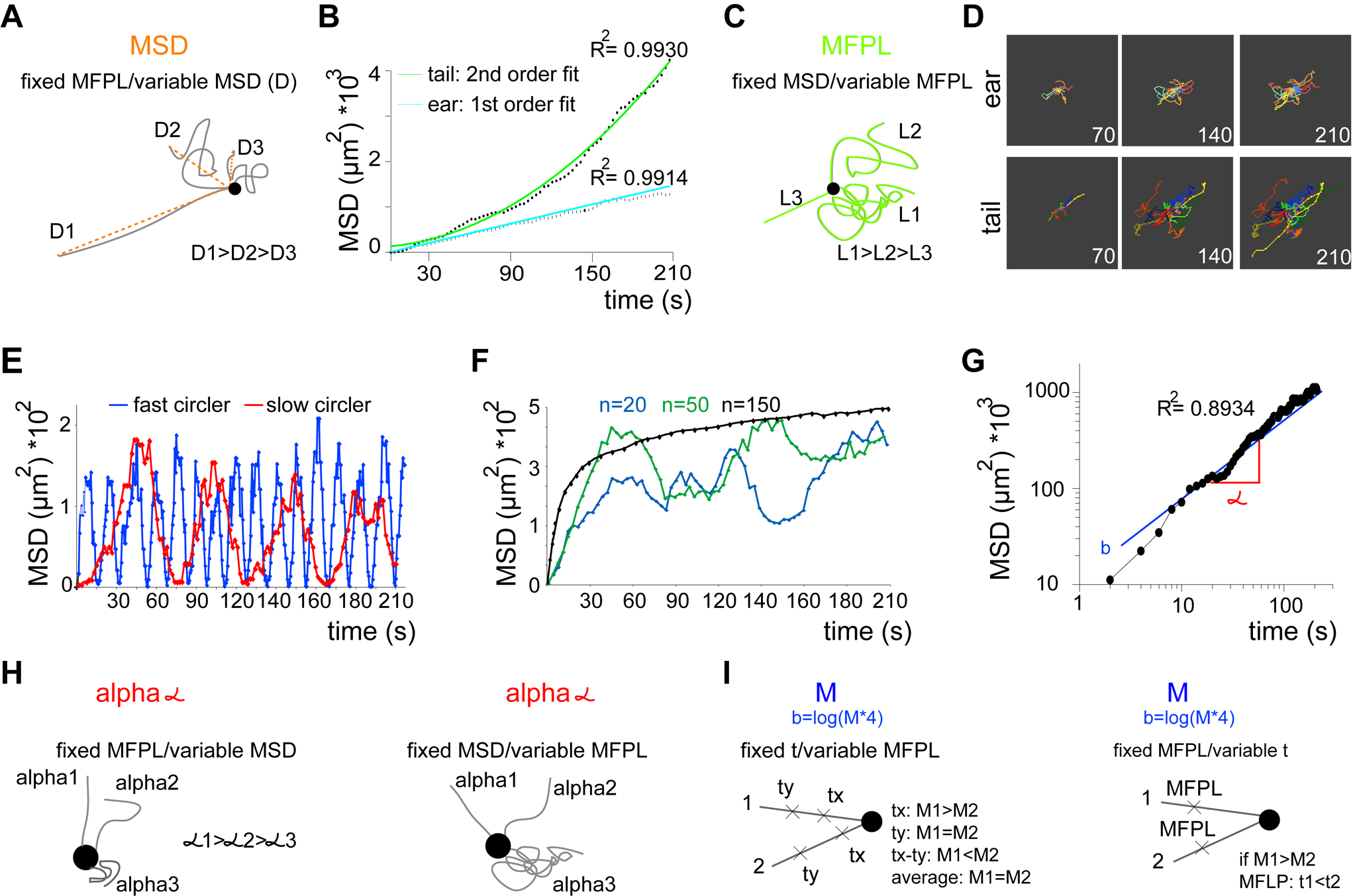

Supplement: Figure S1 — Quantitative analysis of cell and sporozoite displacement. (A) Three different cell trajectories D1, D2 and D3 are shown (grey). The displacement at a certain time point t (D(t) = D) is the direct distance a cell has traveled between the common start point and the coordinates at the time point t (“air line distance”, orange dotted lines). The free path length (FPL, grey lines) is the distance covered by a moving particle for each step from the start point (“real traveled path”). To quantify average displacements and path lengths of a large number of particles/cells the mean square displacement (MSD) and mean free path length (MFPL) are calculated. Particles can cover the same path length (fixed MFPL) but are less far (D3 = lower MSD) or further away (D1 = higher MSD), from the start point (black dot) than D2. (B) 1st order regression or 2nd order polynomial fitting to one data set for parasite migration in the ear or tail showing a very good fitting (R2<0.99). Since linear migration patterns dominate for tail migration over time the regression behaves as 2nd order polynomial. Other patterns predominantly result in first order regressions thus giving a best fitting for migration in the ear where meandering patterns dominate. (C) Cells can be equally far away from the starting point (black dot) (fixed MSD) but have traveled on differently long paths in order to go there (variable MFPL, L3 shorter MFPL than L2 than L1). (D) Track plots of 7 sporozoites migrating in vivo inside the ear and the tail. Individual tracks are shown in different colors and were arranged so they originate at the same x-y position to visualize parasite spread. The absolute time is indicated in seconds. (E) Mean square displacement (MSD) plot over time of two sporozoites turning in circles with higher (blue) and lower speed (red). Individual perfectly circling parasites show no net increase in mean square displacement as they have the same start and end point during one circle. During one circle [file ppat.1002080.s001.tif]

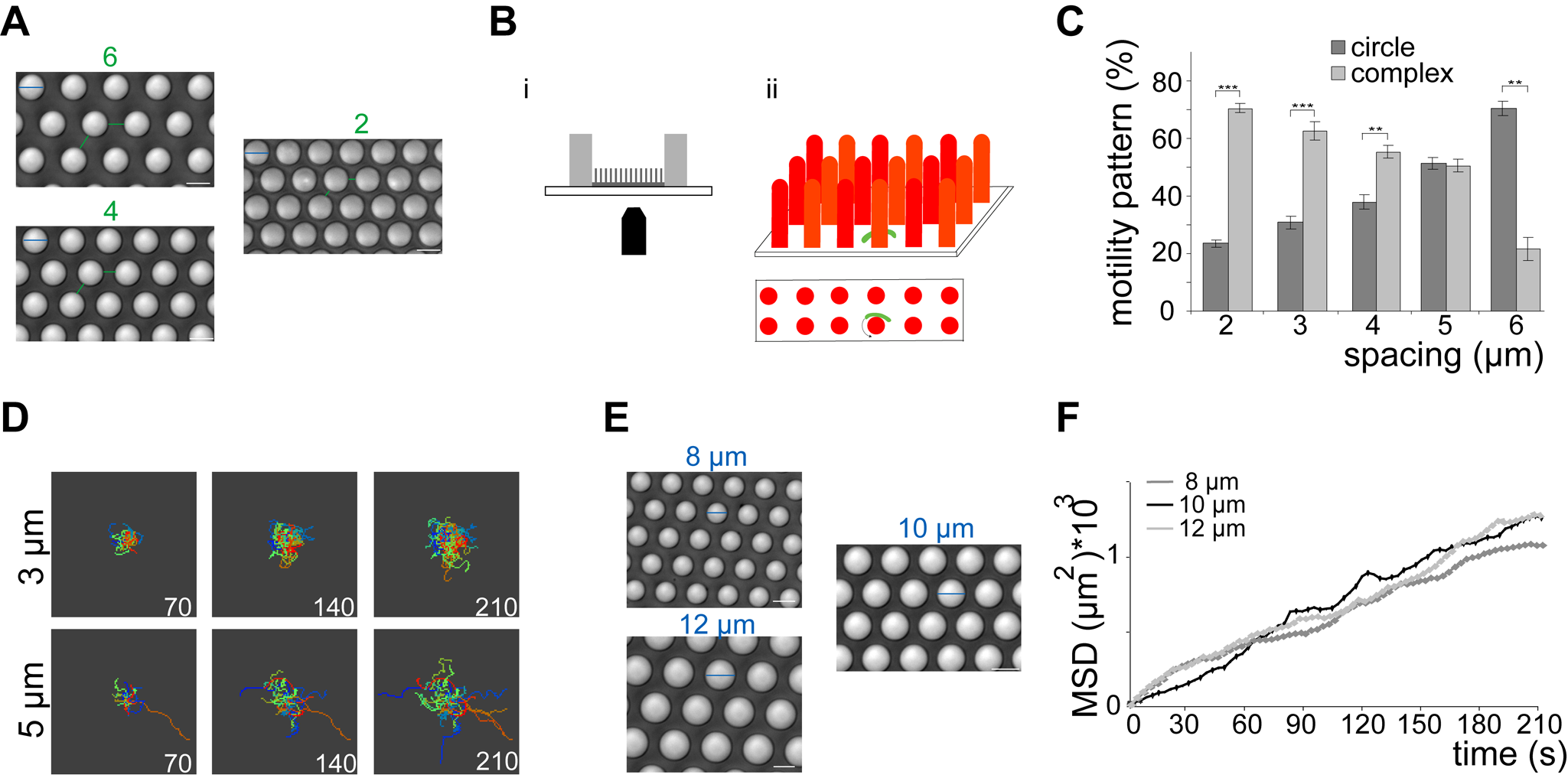

Supplement: Figure S2 — Micro-fabricated obstacle arrays. (A) DIC images of different micro-fabricated obstacle arrays (top view). Pillar diameter (cyan lines) is fixed to 10 µm while pillar-to-pillar distances are varied from 2 to 6 µm (green lines and numbers). Scale bars: 10 µm. (B) (i) Scheme of the experimental setup. The flexiPERM chamber (light grey) surrounds the obstacle array (dark grey) on a glass slide (white) and is imaged with a 10× objective (black). (ii) Cartoon of an obstacle array showing obstacles (red and orange) and a sporozoite (green) in 3D (upper panel) and a top view corresponding to the image in panel A and to Figure 3. The arrow indicates the movement of the sporozoite around a pillar. (C) Manual quantification of sporozoite movement patterns within different obstacle arrays of different pillar-to-pillar spacing. Between 150 and 200 sporozoite trajectories were evaluated for each pattern. All bars show mean ± standard deviation. (D) Track plots of 15 sporozoites migrating in 3 and 5 µm arrays. Individual tracks are shown in different colors and were arranged so they originate at the same x-y position to visualize parasite spread. The absolute time is indicated in seconds. (E) DIC images of different micro-fabricated obstacle arrays (top view). Pillar diameter (blue line) can be varied from 4 to 14 µm (here 8 µm, 10 µm and 12 µm are shown) while pillar-to-pillar distances can be varied from 2 to 7 µm (here 3 µm arrays are shown). Scale bars: 10 µm. (F) MSD over time plot for migrating sporozoites in arrays with different pillar diameters (8; 10 and 12 µm) and pillar-to-pillar spacing fixed to 3 µm. Parasite migration is independent from a pillar diameter between 8 µm to 12 µm. (TIF) [file ppat.1002080.s002.tif]

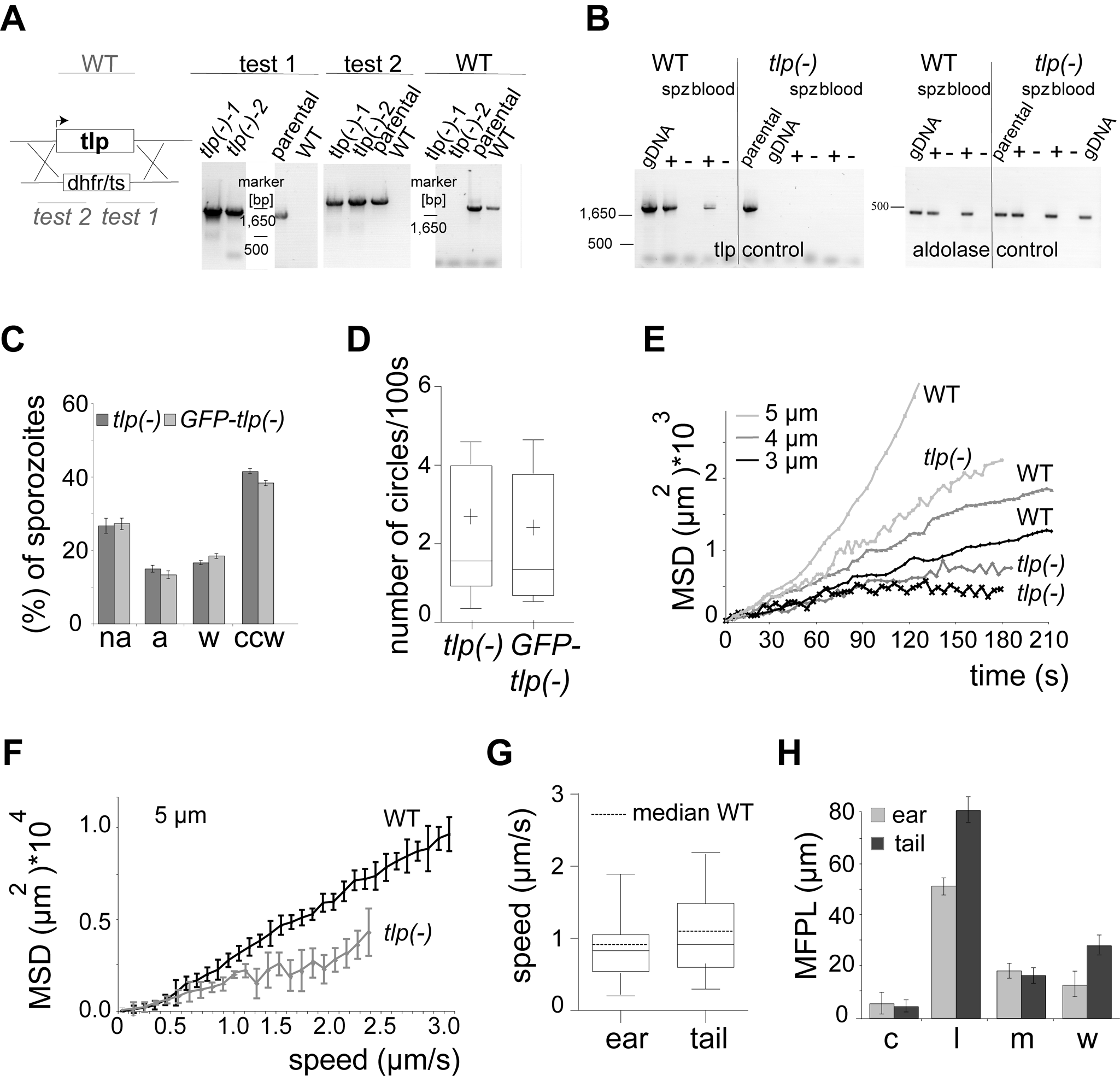

Supplement: Figure S3 — Generation, evaluation and analysis of GFP-tlp(-) parasites. (A) Replacement-specific PCR analysis. In the replacement strategy cartoon the grey lines indicate binding of test primers for WT, tlp and integration of the dhfr/ts gene. PCR analysis reveals the specific disruption of P. berghei tlp locus. The successful replacement event is verified by diagnostic PCR primers [21]. Test 1 and 2 primer combinations can only amplify a signal after successful replacement and integration (see schematic replacement strategy). Absence of the wild type-specific signal from GFP-tlp(-) parasites confirms the purity of the clonal population. tlp(-)1 and 2: recombinant tlp(-) clones; parental: parental line with a mixed population of wild type (WT) and tlp(-) parasites. (B) RT-PCR. Absence of TLP transcripts in tlp(-) parasites. cDNA from WT or tlp(-) late-blood stages (blood) or sporozoites (spz) was amplified in the presence (+) or absence (−) of reverse transcriptase (RT) with PbTLP-specific primer combinations. As loading controls, RT-PCRs with Pbaldolase-specific primers were added. gDNA: genomic DNA from WT or clone tlp(-)-1 parasites. (C) Manual analysis showing that the GFP-tlp(-) sporozoites display the same movement patterns as tlp(-) parasites: na: not attached; a: attached; w: waving; ccw: counter-clockwise moving sporozoites. Note that there are about 10% fewer CCW moving GFP-tlp(-) sporozoites than WT sporozoites. (D) Gliding GFP-tlp(-) sporozoites move in the same manner as tlp(-) sporozoites. Analysis was performed as described in [19]. (E) MSD plots of WT and GFP-tlp(-) sporozoites moving in obstacle arrays of 3, 4 and 5 µm pillar-to-pillar distance. (F) MSD plotted over average sporozoite speed of WT and GFP-tlp(-) sporozoites moving in obstacle arrays of 5 µm pillar-to-pillar distance, respectively. (G) Speed plot showing the median speed distribution of all tracked GFP-tlp(-) parasites imaged in the dermis of ear or tail. The dotted line marks the WT median valu [file ppat.1002080.s003.tif]

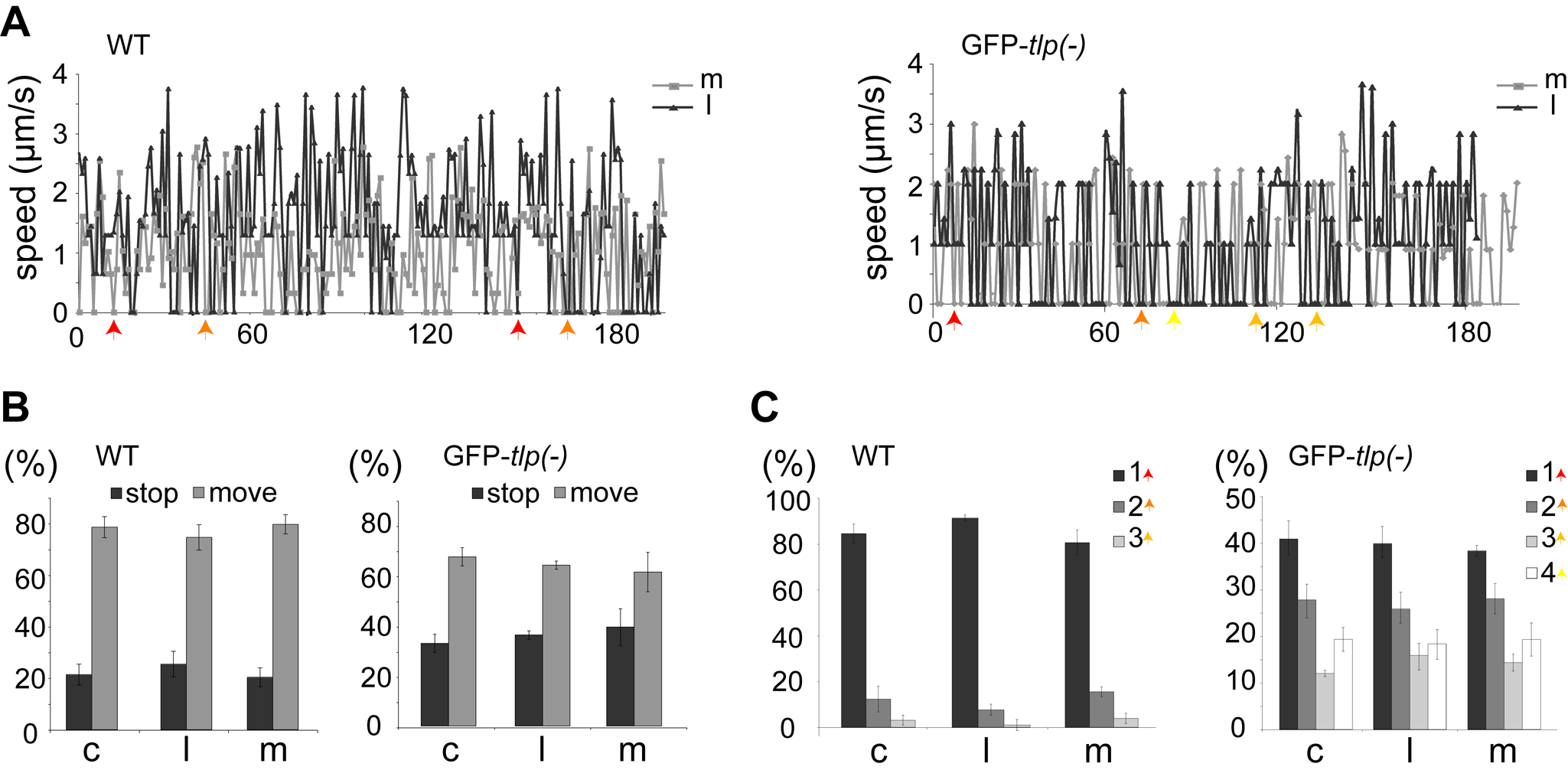

Supplement: Figure S4 — Stop and Go phases of parasite migration in obstacle arrays. (A) Example of single WT and tlp(-) sporozoites moving in a meandering (m) or linear (l) pattern. Speed (µm/s) is plotted over time. To analyze Stop phases all time points with parasite speeds lower than 0.3 µm/s were scored. The arrows point on exemplary Stop phases with a duration of 1 (red), 2 (orange), 3 (light orange) or 4 (yellow) frames. These durations were added up for the plots in (B and C). (B) Quantitative analysis of wild type or tlp(-) parasites turning in circles (c, n = 12/11), moving linear (l, n = 14/15) or meandering (m, n = 16/15) are plotted in respect to the total percentage of Stop (stop) and Go (move) phases. (C) Quantitative analysis of the duration for a stop phase as measured in time-frames from 0.5 Hz image acquisitions and plotted for WT and tlp(-), respectively. (TIF) [file ppat.1002080.s004.tif]
