# Supplementary material for: Environmental Constraints Guide Migration of Malaria Parasites during Transmission
Source: PLoS Pathog. 2011 Jun 16;7(6):e1002080. doi: 10.1371/journal.ppat.1002080 (PMC3116815; doi:10.1371/journal.ppat.1002080)
Supplement: Table S1 — Movement patterns listed according to environment. Non moving parasites (∼20% in arrays, ∼30% in vivo) make up the missing percentage to 100%. (DOC) [file ppat.1002080.s005.doc]

**Table S1** Movement patterns listed according to environment

| **Condition** | **Circle (%)** | **Linear (%)** | **Meandering (%)** |
| --- | --- | --- | --- |
| **3 µm** | 23 | 8 | 50 |
| **4 µm** | 31 | 12 | 40 |
| **ear** | 10 | 10 | 48 |
| **5 µm** | 42 | 31 | 12 |
| **tail** | 11 | 32 | 26 |
